# Computational Design of 2D Nanoporous Graphene via Carbon-Bridged Lateral Heterojunctions in Armchair Graphene Nanoribbons

**DOI:** 10.1021/acsomega.4c07524

**Published:** 2025-04-23

**Authors:** Rodrigo A. F. Alves, Kleuton A. L. Lima, Daniel A. da Silva, Fábio L. L. Mendonça, Luiz A. Ribeiro Junior, Marcelo L. Pereira Junior

**Affiliations:** † Institute of Physics, University of Brasília, Brasília 70910900, Federal District, Brazil; ‡ Computational Materials Laboratory, LCCMat, Institute of Physics, University of Brasília, Brasília 70910900, Federal District, Brazil; § Professional Postgraduate Program in Electrical Engineering (PPEE), Department of Electrical Engineering, College of Technology, University of Brasília, Brasília 70910900, Federal District, Brazil; ∥ College of Technology, Department of Electrical Engineering, University of Brasília, Brasília 70910900, Federal District, Brazil; ⊥ Materials Science and NanoEngineering, Rice University, Houston, Texas 77005, United States

## Abstract

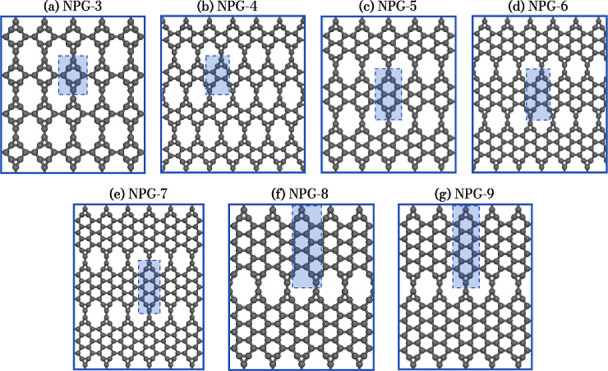

The interest in two-dimensional (2D) carbon allotropes arises from their ability to alter their properties based on the atomic topology employed, which can significantly affect their electronic properties and benefit advancements in new technologies. This work presents a new nanoporous graphene (NPG) allotrope obtained through lateral heterojunctions via pairs of trivalent sp^2^ carbon atoms of armchair graphene nanoribbons (AGNRs). These pairs were used as linkers between AGNRs to achieve this structure, forming connections that enhance the porous architecture. This novel planar and porous 2D carbon allotrope integrates some structural and electronic advantages of AGNRs into a 2D framework. Composed of 3-, 6-, and 12-membered carbon rings, the NPG was investigated using density functional theory (DFT) calculations and ab initio (AIMD) and classical molecular dynamics (CMD) simulations to explore its structural, electronic, and mechanical properties. Among the results presented, we show that the material demonstrates high dynamical and thermal stability at 1000 K. Furthermore, the NPG exhibits metallic and nonmagnetic behavior and is achieved by transitioning from the semiconducting nature of some AGNRs to a metallic 2D carbon system. The elastic properties reveal the material’s distinct response to applied strain, with fractures occurring in the nanoribbon segment along the *x*-direction. However, fractures are observed in the C–C bonds involved in the heterojunction region in the *y*-direction. The calculated Young’s modulus ranges from 394 to 690 GPa, which is lower but comparable to graphene. The formation energy of NPG decreases with increasing width of the AGNRs used to compose the 2D material, indicating enhanced stability for wider nanoribbons. These findings highlight the potential of NPG for applications in nanoelectronics and advanced new technologies.

## Introduction

Exploring 2D carbon allotropes has become essential due to their attractive structural and electronic properties, which have revolutionized fields such as planar electronics.^1,2^ Unlike their one-dimensional and three-dimensional counterparts, these 2D materials, often a single atomic layer thick, exhibit characteristics enabling innovative applications across various technologies.^3,4^ Their finely tunable atomic structure, mainly due to their area-to-volume ratio, makes them ideal candidates for advancements in electronics and materials science.^5,6^

Recent developments have introduced a variety of new 2D carbon allotropes, expanding the possibilities for their application in fields ranging from biomedical engineering,^7^ energy storage,^8^ green energy,^9^ optoelectronics,^1^ and others.^10^ To enable these applications, the theoretical prediction of new two-dimensional systems has constantly developed to advance new nanomaterials and their possible applications.^11^ It also drives the search for synthesis routes for these systems.^12^ In a recent scenario, carbon allotropes—in addition to graphene^13,14^ and γ-graphyne,^15,16^ which were theoretically predicted before their syntheses—such as ψ-graphene,^17,18^ biphenylene network,^19,20^ holey-graphyne,^15,21^ and fullerene network^22,23^ are examples of two-dimensional nanomaterials that have been synthesized, and their theoretical predictions were already known.

A critical and emerging trend in developing 2D carbon materials is the creation of porous structures. These porous 2D allotropes offer enhanced functionality for various applications,^24^ including gas sensors,^25^ catalysis,^26^ environmental remediation,^27^ desalination,^28^ and water treatment.^29^ With their high surface area and adjustable pore sizes, these systems provide significant advantages in capturing and converting small molecules such as CO_2_ and facilitating catalytic reactions.^30^ Integrating these porous features into 2D structures opens new avenues for addressing pressing global challenges such as environmental pollution and energy storage. However, new systems and methodologies require attention from the scientific community to obtain and understand these porous materials.

In this perspective, Moreno et al. performed the bottom-up synthesis of a nanoporous graphene monolayer from the monomer DP-DBBA. A polymerization followed by cyclo-dehydrogenation resulted in a 13-AGNR nanoribbon with well-defined cove-type structures, forming a 7-13-AGNR heterojunction. In a final step, dehydrogenative cross-coupling enabled the lateral joining of these nanoribbons, thus synthesizing multifunctional nanoporous graphene with a pore size of around 1 nm.^31^ The authors discuss various potential applications of the synthesized nanomaterial. However, the literature has not yet reported lateral heterojunctions of graphene nanoribbons without cove-type structures on the edges.

This work introduces a class of NPGs obtained from the lateral heterojunction of AGNRs of different widths (*n* = 3, ..., 9), integrating the structural and electronic advantages of AGNRs into a nanoporous 2D structure. Featuring a flat structure with 3-, 6-, and 12-membered rings, the NPG combines high stability with unique electronic and mechanical properties. By exploring its formation and deformation characteristics through DFT, AIMD, and CMD calculations, this work aims to demonstrate the potential of this class of NPGs as a resilient and versatile material, contributing to the design of innovative flat technologies.

## Methodology

In our studies, a quantum approach was employed to obtain the structural characteristics, stability, and electronic structure of NPG. Conversely, a classical methodology was utilized to determine the mechanical properties of the nanomaterial under the influence of temperature.

### DFT Calculations

To investigate the structural characteristics, stability, and electronic behavior of the NPG, simulations based on DFT were conducted using the Vienna Ab initio Simulation Package (VASP),^32^ where the Kohn–Sham (KS) equations were solved using the projector augmented wave (PAW) method,^33^ with plane-wave cutoff energy set at 2 × *E*_max_, where *E*_max_ is the maximum recommended cutoff energy for systems containing only carbon atoms.^33,34^ The calculations for the NPG structures were performed using the generalized gradient approximation (GGA),^35^ with the Perdew–Burke–Ernzerhof (PBE) exchange–correlation functional.^36^ A 0.01 eV/Å criterion was used to minimize the stress tensor and atomic forces. After optimizing the NPGs under investigation, the remaining electronic properties were obtained with a cutoff energy of 1.25 × *E*_max_. A total energy convergence criterion of 10^–6^ eV was adopted to achieve a self-consistent electron density. To eliminate any interaction between the monolayer and its periodic images in the *z* direction, a vacuum space of 15 Å was included in each NPG unit cell. For all calculations, the *k*-meshes were automatically generated using the Monkhorst–Pack method,^37^ ensuring a *k*-point density of 40 Å^–1^ in the in-plane lattice vector directions. This procedure ensures consistency in the calculations of the electronic properties and band structure of the NPG and is a well-established process in the literature.^38^ To verify the dynamic stability of the nanomaterial, a 3 × 3 × 1 supercell was used to obtain the phonon dispersion, with the same criteria as defined above. To verify the thermal stability of the NPG, AIMD calculations were performed on all systems using the *NVT* ensemble, with temperatures up to 1000 K, a time step of 1 fs, for a total of 5 ps.

### CMD Calculations

To investigate the mechanical properties of the NPG, stress–strain simulations were conducted using classical molecular dynamics with the LAMMPS (Large-scale Atomic/Molecular Massively Parallel Simulator) software.^39,40^ The simulations employed the velocity-Verlet algorithm^41,42^ to integrate Newton’s equations of motion with a time step of 0.1 fs. Before applying the deformation, the intrinsic stresses of the system were eliminated by allowing the systems to evolve using the *NPT* ensemble for 250 ps, with zero pressure and room temperature. The system was then subjected to an additional 250 ps simulation with room temperature and the *NVT* ensemble for thermalization of the nanomaterial. During the deformation tests, the nanoribbons’ elastic properties and fracture patterns were evaluated at a temperature of 300 K, with the NPG being deformed at a constant rate of 10^–6^ fs^–1^. The AIREBO (Adaptive Intermolecular Reactive Empirical Bond Order) interatomic potential^43^ was used to characterize bond breakages and formations. This potential has been applied to various other similar carbon systems. Since NPG is a lateral junction AGNRs, this potential is suitable for describing the system’s atomic interactions, even though approaches using machine learning interatomic potentials are also commonly employed in studying these systems.^44^ Moreover, it is known that adjusting the cutoff radius of AIREBO is necessary for a better description of the mechanical response of the NPG. Deformation was considered separately in the *x* and *y* directions, using the isobaric–isothermal ensemble, to avoid additional stresses in the perpendicular directions. The influence of NPGs deposited on a gold substrate on their mechanical properties was also investigated, and the details are in Section SI of the Supporting Information.

Except for the deformation applied to the systems during the investigation of mechanical properties, the same system preparation protocol was followed, using the *NPT* ensemble to eliminate external stresses and the *NVT* ensemble to thermalize the system. In addition to the previously discussed baseline parameters, the systems were subjected to a temperature ramp from 300 to 8000 K at a rate of 15.4 K/ps to assess their thermal stability. The simulation details are presented in Section SII of the Supporting Information.

### System Modeling

**Figure 1:**
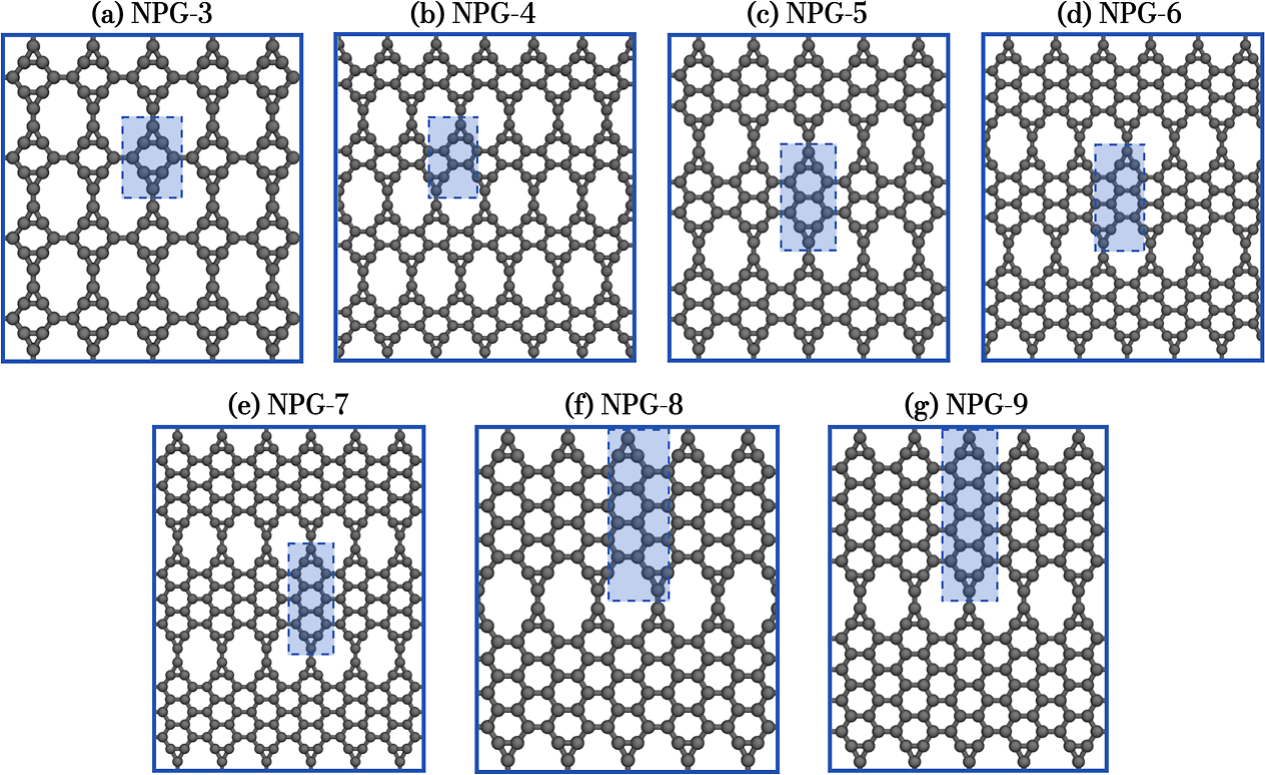
NPG systems investigated, with lateral junctions of AGNRs. The rectangles highlighted inside each panel represent the unit cell of each system.

This study proposes the structural, electronic, and mechanical investigation of nanoporous graphene membranes, employing quantum and classical methodologies. For the construction of the system, AGNRs with widths ranging from 3 to 9 carbon atoms were considered (see Figure 1). Pairs of trivalent sp^2^ carbon atoms were introduced between the hexagonal rings to form triangular linkages. This strategy was adopted to increase the nanopore size, as linking all atoms with open valence at the nanoribbon edges would result in lower porosity. Consequently, the NPG structures considered here comprise 3-, 6-, and 12-membered carbon rings, with the pore density decreasing as the AGNR width increases. Figure 1 schematically represents all NPGs considered in this study. The pores are paired for NPG-*n* structures with even *n*, while those with odd *n* show an unpaired arrangement. Additionally, all systems exhibit sp^2^ hybridization.

## Results and Discussion

### Structural Properties

Figure 1 illustrates the atomic structure of NPGs, highlighting the arrangement of carbon atoms and unit cells. Panels (a,c,e,g) show the structure with an odd number of carbon atoms across the width of the AGNR, while panels (b,d,f) display an even number of carbon atoms across the width of the nanoribbon. Due to changes in symmetry, the unit cells of NPG-4, -6, and -8 have relatively higher numbers of atoms in the unit cells. These configurations reflect different structural characteristics of the 2D material proposed in this study. NPGs feature a lattice comprising 3-, 6-, and 12-membered carbon rings, creating a distinct pattern within their unit cells, as illustrated in Figure 1 by the blue rectangles. The unit cells contain 8, 12, 16, and 20 atoms for the odd widths, with *n* = 3, 5, 7, 9, and have dimensions of approximately 4.42 Å in the *x* direction across all cases, while in the *y* direction, the values are 6.09, 8.57, 11.06, and 13.53 Å, respectively. For the NPG-*n* monolayers with even *n*, the number of atoms per unit cell is 20, 28, and 36, with dimensions of 14.69, 19.64, and 24.60 Å in the *y* direction for *n* = 4, 6, 8, respectively. The unit cell width is approximately the same for both even and odd *n*. The C–C bond lengths in NPG were measured as 1.43 Å for the bonds within hexagons, 1.37 Å for the bonds connecting hexagonal and triangular rings, 1.42 Å for the bonds exclusive to triangular rings, and 1.35 Å for the bonds connecting the triangular rings. The bond lengths in the hexagonal regions (along the AGNRs) have their cores ranging from 1.42 to 1.45 Å. The bonds parallel to the longitudinal direction of the nanoribbons are slightly elongated, around 1.45 Å. The bonds oblique concerning the longitudinal direction are around 1.43 Å. In contrast, at the edges of the nanoribbons, the bonds (oblique concerning the longitudinal direction) are 1.39 Å at the edges of the nanoribbon concerning the hexagons involved with the carbon bridge and 1.42 Å for the bonds parallel to the longitudinal directions, for hexagons not involved in the linker between the AGNRs.^45,46^

**Figure 2:**
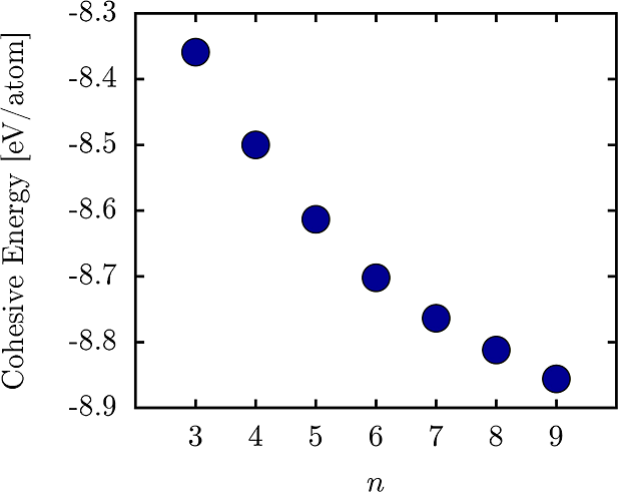
Calculated cohesive energy of NPG as a function of the AGNR width (*n*).

The crystal structure of the NPG-*n* family investigated here is classified under the *PMMM* (*D*_2H_^1^) symmetry group for even *n* and *CMMM* (*D*_2H_^19^) for odd *n*, both within the orthorhombic system. The cohesive energy was calculated from the expression *E*_coh_(*n*) = (*E*_NPG-*n*_ – *n*·*E*_C_)/*n*, as a function of the ribbon width *n* of the AGNRs, and is presented in Figure 2. It can be observed that the formation energy of NPG-*n* decreases for wider ribbons, reaching approximately −8.9 eV/atom, indicating good energetic stability. This decreasing trend is expected to approach the cohesive energy of graphene, which was calculated here to be around −9.2 eV/atom. These values are comparable to other 2D carbon allotropes, being lower than those of holey-graphyne (−7.3 eV/atom),^21^ BPN (−7.4 eV/atom),^47^ and irida-graphene (−7.0 eV/atom),^48^ as well as spanning the range (−8.3 to −8.9 eV/atom) of several other carbon allotropes reported in the literature.^17,49,50^ These values indicate the stability of NPG-*n* within 2D carbon materials. Furthermore, the crystal lattice of NPG remains planar, with no observed buckling, across all AGNR widths studied.

**Figure 3:**
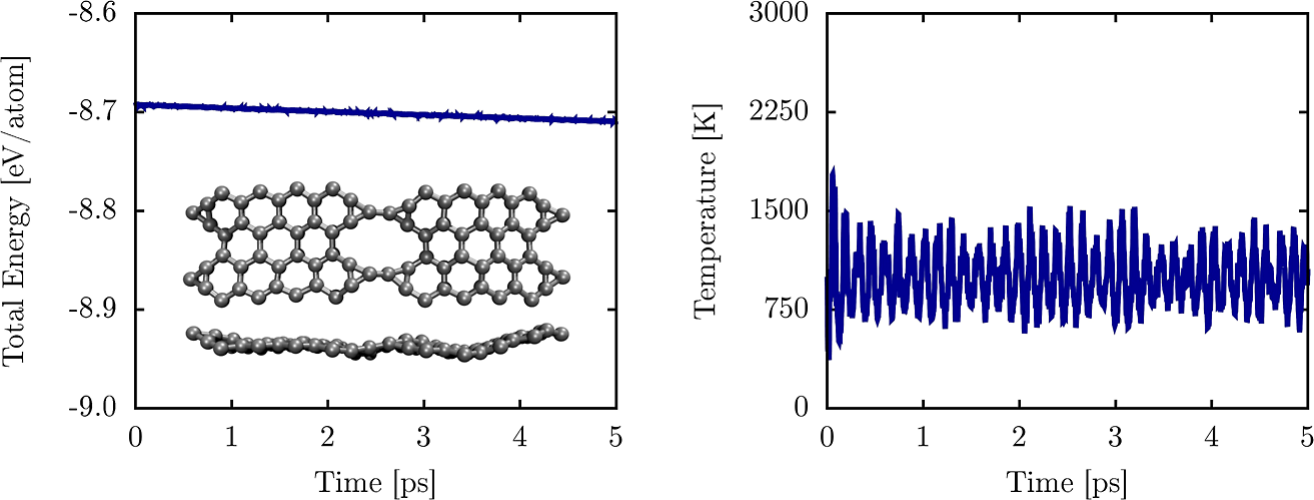
Left panel depicts the temporal evolution of the total energy per atom lattice at 1000 K. The inset illustrates top and side views for THD-graphene (case *k* = 9) in this panel at 5 ps. The right panel illustrates the system temperature over the simulation period.

To dynamically confirm the stability of the NPGs, Figure 3 presents AIMD simulations to evaluate the thermal stability of the nanomaterial at 1000 K. As a representative case, we show the results for the system with *n* = 9. It is worth noting that the other NPG cases exhibited similar AIMD results, with shifts in the average energy consistent with Figure 2. Therefore, they are not displayed here. The left panel shows the temporal evolution of the total power over the 5 ps simulation. The total energy profile remains constant, with minimal fluctuations, indicating that NPG-9 maintains thermal stability under these conditions.

The inset panels in Figure 3 provide top and side views of the final snapshot from the AIMD simulation. Although some lattice deformation is observed at 1000 K, no bond breakage occurs, and the material retains its overall structural integrity. The observed deformations are primarily attributed to slight changes in planarity and minimal variations in bond distances, typical under thermal stress. These results confirm that NPG-9 exhibits good dynamical stability at elevated temperatures. The right panel of Figure 3 illustrates the time evolution of the system’s temperature. The average temperature stabilizes around 1000 K, indicating effective thermal regulation throughout the simulation. The same behavior is observed for all NPGs discussed in this study.

In addition to the AIMD simulations, where we verified the thermal stability up to 1000 K, we also performed CMD simulations. These CMD calculations applied a temperature ramp during a 500 ps simulation, with a heating rate of 15.4 K/ps applied to the thermal bath for all the NPGs studied here. The details of these calculations are provided in Section SII of the Supporting Information. Figure S2 illustrates the energy evolution of the systems as a function of temperature, along with the normalized heat capacity. Based on these results, we demonstrated that the main phase transition point of the NPGs does not depend on the ribbon width and occurs at a critical temperature of 2338 K.

**Figure 4:**
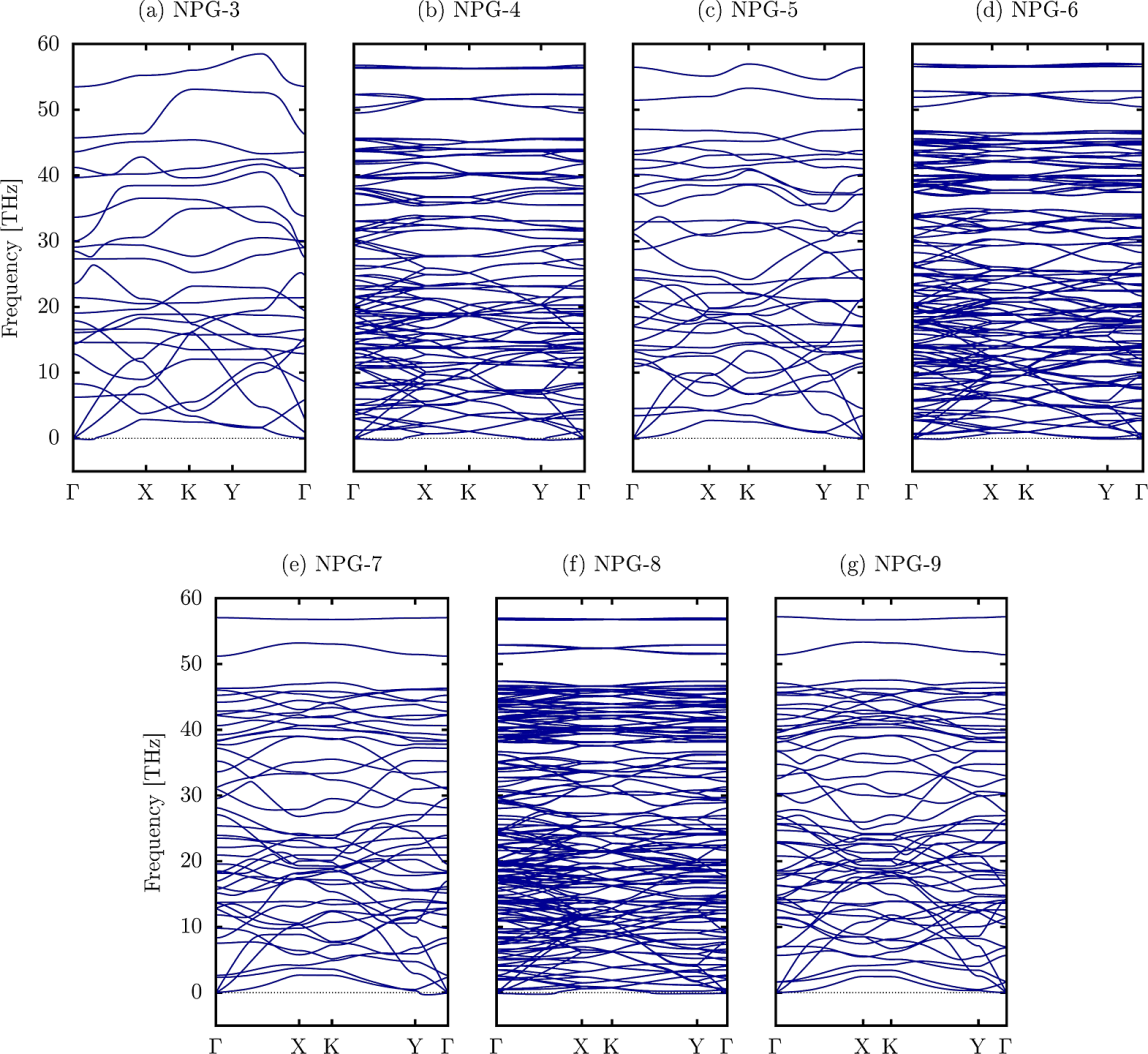
Phonon dispersion curves of NPG materials.

The phonon dispersion curves of NPG-*n*, shown in Figure 4, provide crucial insights into their dynamic stability and lattice vibrations of the nanomaterials investigated herein. In panels 4c,e,g, the absence of imaginary frequencies throughout the Brillouin zone indicates that these NPG structures are dynamically stable. This result confirms that these structures are free from mechanical instabilities, making them promising for practical applications, as previously discussed in Figures 2 and 3. In contrast, Figure 4a,b,d,f display minor imaginary frequencies. These frequencies suggest the presence of intrinsic stress within the material but do not imply system instability, as their magnitudes are around 0.1 THz and can be easily alleviated with minor biaxial stress applied to the nanomaterials.^51^ The structures remain stable despite these minor imaginary frequencies, as previously mentioned and confirmed by both cohesion energy and AIMD calculations. This stability, even with intrinsic stress, is a significant attribute for practical applications, indicating that the material can withstand certain degrees of deformation without losing its structural integrity.

Graphene and AGNRs stand out for their unique phononic properties, which have been extensively studied.^52,53^ These materials are characterized by high-frequency optical modes and a clear separation between acoustic and optical branches. In NPG materials, the highest phonon frequency is approximately 59 THz, slightly higher than the 49.11 THz observed in pristine graphene.^54^ This slight increase can be attributed to the unique combination of 3-6-12 membered rings in the NPG topology, which introduces additional constraints on atomic vibrations compared to the purely hexagonal graphene lattice. The presence of these rings likely contributes to the observed increase in phonon frequency.

Figure 4 shows that it is observed that the phonon spectra of NPG do not display a distinct gap between the acoustic and optical modes. This feature implies a finite scattering rate between these modes, potentially leading to shorter phonon lifetimes. Such behavior is indicative of moderate lattice thermal conductivity. Understanding these phonon lifetimes and scattering rates is crucial for materials intended for thermal management applications, as they directly influence the material’s ability to conduct heat.

The overall phonon behavior in NPG reflects the intrinsic properties of its GNR constituents. However, the fusion of different ring sizes introduces novel characteristics. The even-width nanoribbons, in particular, significantly enhance the material’s vibrational stability and dynamic integrity by populating the Brillouin zone with more phonon modes than the odd-width cases, potentially improving its suitability for thermal management applications.

### Electronic Properties

**Figure 5:**
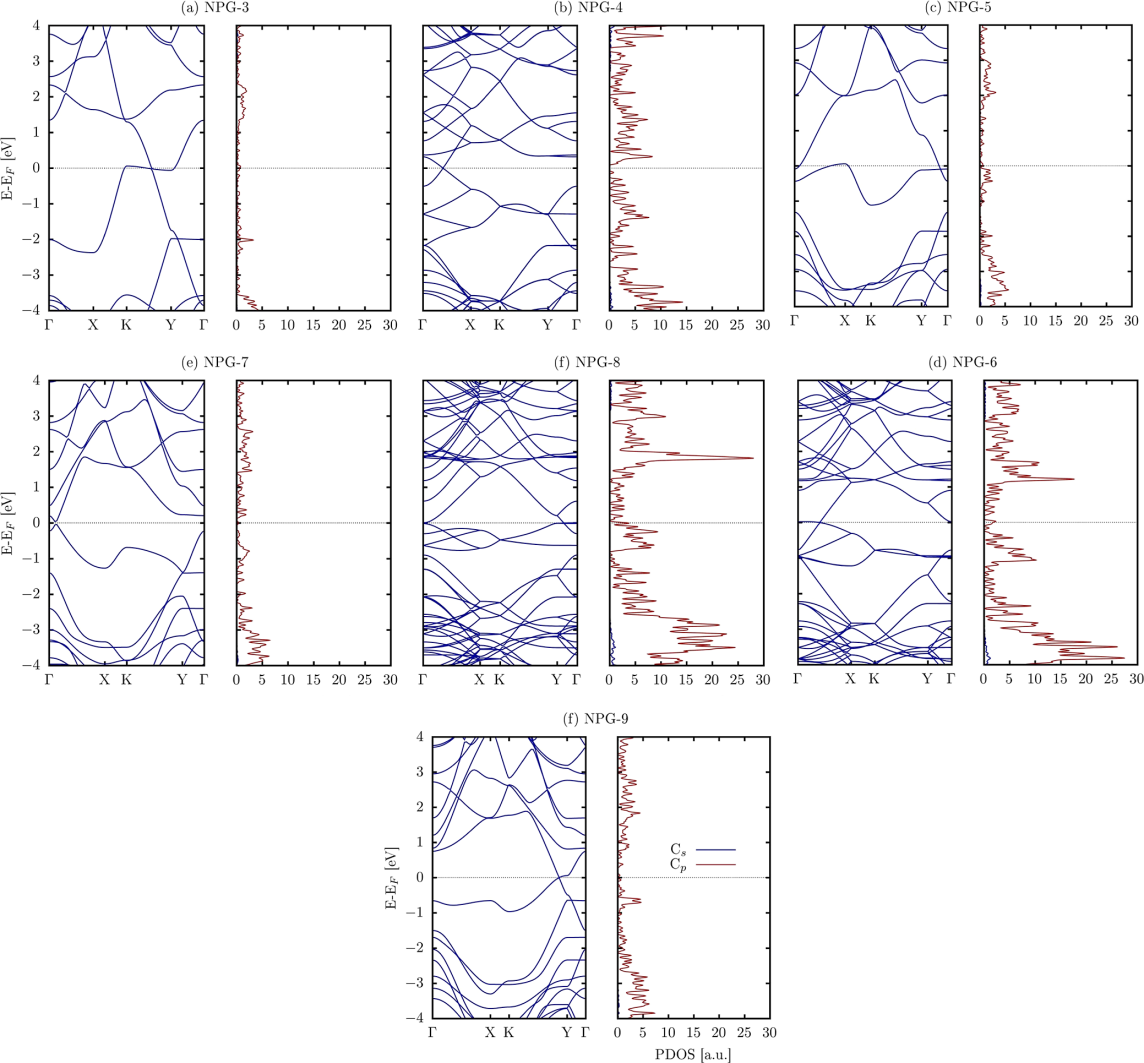
Electronic band structure and related partial density of states (PDOS) for the NPG cases. The calculated PDOS of atoms concerning s and p orbitals.

We now turn to the electronic band structures of the NPG-*n* systems, as illustrated in Figure 5, which provide insights into their conductive behavior. The results consistently show the absence of a band gap, characterizing the NPG as metallic. This metallic nature suggests that the material has excellent potential for efficient charge transport, similar to free-electron behavior. The anisotropic conductance observed in the band structures indicates semiconducting behavior along specific crystallographic directions, specifically Γ–*K*, while maintaining metallic characteristics along others, such as *K*–*Y* and *Y*–Γ. This anisotropy in electronic properties could be leveraged for directional conductivity control in various applications, particularly in nanoelectronics, where specific directional conductance can be advantageous.

The projected density of states (PDOS) provides further electronic structure analysis. The PDOS analysis reveals a dominant contribution from p-states. This predominance of p-orbitals, known for their directional bonding, is critical in shaping the electronic properties of the NPG systems. The negligible contribution of s-states highlights the pivotal role of p-orbitals in this material’s electronic behavior. The PDOS findings corroborate the metallic nature observed in the band structure analysis.

A comparison of the electronic properties of the NPG family investigated here with those of graphene and graphene nanoribbons is also insightful. Graphene, known for its remarkable electronic properties, such as high carrier mobility and the presence of Dirac cones,^55,56^ shares similarities with NPG in terms of p-orbital dominance and metallicity. Like graphene, which exhibits a Dirac cone at the Fermi level, NPG also shows this feature, emphasizing its electronic parallel with graphene. In addition to the absence of a band gap, NPG systems can exhibit intriguing features such as Dirac cones slightly tilted above the Fermi level. This tilt has been observed in other Dirac/Weyl materials and indicates systems where the effective spacetime is non-Minkowskian and deformed.^57−59^ This deformation can lead to unique electronic behaviors and highlights the importance of further studying NPG, potentially opening new avenues for research and applications in quantum materials and devices. These results show that the presence of nanopores in graphene does not significantly alter its electronic behavior. This indicates that dimensionality is a more relevant factor, as AGNRs exhibit electronic behavior mainly governed by their width and edge type.^60^

### Mechanical Properties

**Figure 6:**
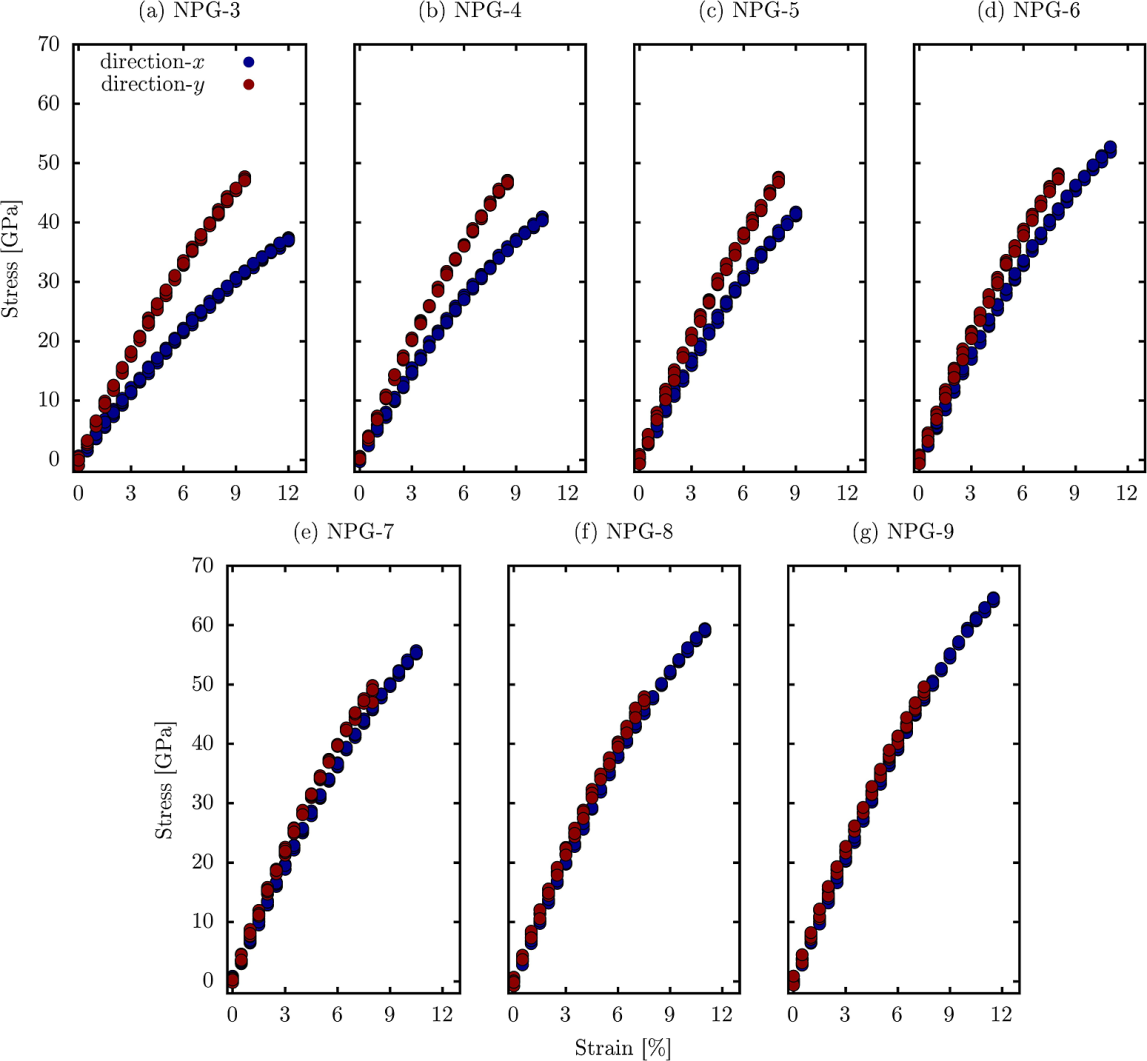
Stress–strain curves for the NPG systems with uniaxial strain in the *x*-direction (blue) and *y*-direction (red).

Finally, the mechanical properties of the NPG lattices, investigated through classical molecular dynamics simulations, provide essential insights into their behavior under applied stress and, consequently, their potential applications. Figure 6 illustrates the stress–strain curves for various NPG lattices studied here. These results show that NPG tends to be less resistant to stress applied in the *x* direction, which is parallel to the length of the AGNR. This trend can be attributed to the influence of the most rigid part of the lattice, composed of fused hexagonal rings, triangular rings with no degrees of freedom for movement, and the strong bonding provided by the triangular–triangular bond. Another essential observation from the stress–strain curves is that as the nanoribbon’s width increases, the system’s degree of anisotropy in response to applied stress in different planar directions decreases. This behavior occurs because wider nanoribbons exhibit mechanical properties that are more similar to those of graphene. Table 1 summarizes the elastic properties derived from these curves. The calculated Young’s modulus for NPG ranges from 394 to 690 GPa, which, while lower than graphene’s approximately 1 TPa,^61^ still indicates substantial stiffness. For narrower AGNRs, the mechanical response reveals distinct fracture patterns depending on the direction of the applied strain. It is important to mention that we evaluated the stress–strain behavior of NPGs deposited on a gold substrate (see Section SI of the Supporting Information), and both the gas phase systems and those deposited on the substrate exhibit the same mechanical response characteristics under tensile loading.

**Table 1:** Mechanical Properties of NPG-*n* with Applied Strain in the *x* and *y* Directions

*n*	*x*-direction			*y*-direction		
	*Y*_M_ (GPa)	σ_C_ (GPa)	ε_C_ (%)	*Y*_M_ (GPa)	σ_C_ (GPa)	ε_C_ (%)
3	394.4 ± 2.3	37.7 ± 0.3	12.3 ± 0.1	619.3 ± 4.5	48.4 ± 1.0	9.8 ± 0.3
4	498.7 ± 8.5	42.1 ± 0.4	11.1 ± 0.3	693.0 ± 7.6	49.1 ± 1.1	9.0 ± 0.3
5	557.6 ± 2.9	42.2 ± 0.3	9.3 ± 0.1	718.2 ± 8.7	49.7 ± 1.2	8.7 ± 0.3
6	595.1 ± 12.0	53.9 ± 0.8	11.6 ± 0.4	729.1 ± 4.1	49.5 ± 0.8	8.4 ± 0.1
7	650.9 ± 5.1	56.6 ± 0.1	10.9 ± 0.4	756.7 ± 9.4	50.0 ± 0.5	8.1 ± 0.1
8	665.8 ± 9.5	61.0 ± 0.7	11.6 ± 0.2	754.2 ± 10.5	49.8 ± 1.3	7.9 ± 0.2
9	685.8 ± 11.2	65.3 ± 0.8	11.8 ± 0.2	758.1 ± 9.5	50.6 ± 1.1	7.9 ± 0.2

**Figure 7:**
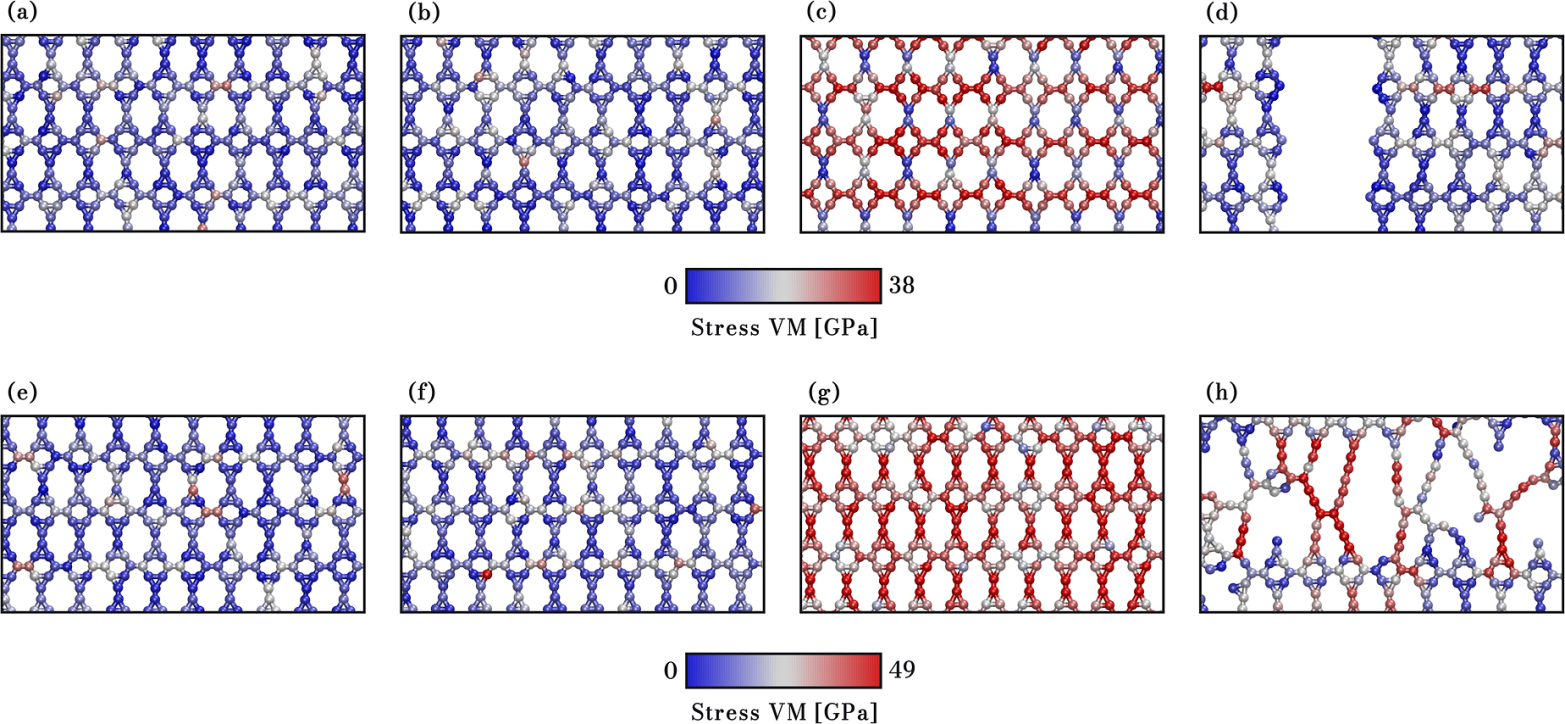
Snapshots of NPG deformation (case *n* = 3, the narrower AGNR studied here) in the *x*- and *y*-directions. Panels (a) to (d) illustrate deformations of 0%, 1.0%, 12.0%, and 12.4% in the *x*-direction, respectively, while panels (e) to (h) depict deformations of 0%, 1%, 9.0%, and 9.8% in the *y*-direction, respectively. Atom color code corresponds to von Mises stress.

**Figure 8:**
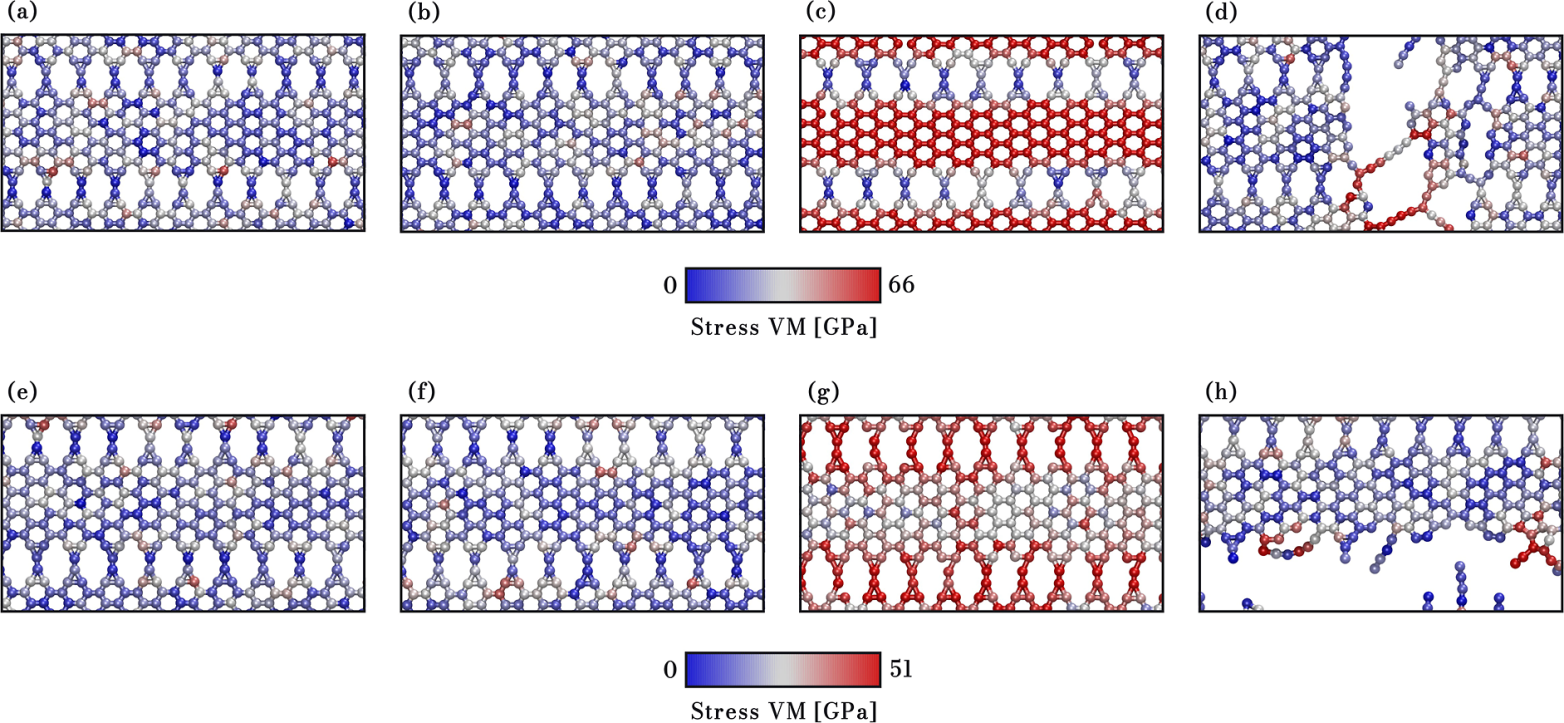
Snapshots of NPG deformation (case *n* = 9, the thicker AGNR studied here) in the *x*- and *y*-directions. Panels (a) to (d) illustrate deformations of 0%, 1.0%, 11.8%, and 12.1% in the *x*-direction, respectively, while panels (e) to (h) depict deformations of 0%, 1%, 7.5%, and 7.9% in the *y*-direction, respectively. Atom color code corresponds to von Mises stress.

As a general trend, fractures in the *x* direction typically occur in the segment of the nanoribbon, as illustrated in Figure 7. In contrast, fractures in the *y* direction are observed in the C–C bonds of the heterojunction region. This fracture behavior also occurs in thicker AGNRs (see Figure 8). Figure 7a–d and e–h present MD snapshots showing the fracture process under *x*- and *y*-directional stress at 300 K. These snapshots use a color scheme representing von Mises (VM) stress per atom,^62−65^ where red indicates high-stress regions and blue indicates low-stress areas. The VM stress values are crucial for understanding fracture initiation and propagation.

When subjected to tensile loading in the *x* direction, the NPG-3 lattice remains intact up to a critical strain value. For example, at 0%, 1%, and 12.0% strain, the structure shows no failure (Figure 7a–c). However, beyond the critical strain (e.g., 12.4%), the material undergoes a sudden transition from elastic deformation to complete fracture (Figure 7d). The crack propagates rapidly in the *y* direction, opposite the tensile loading direction, with the C–C bonds between the hexagons parallel to the applied stress direction accumulating the most stress and breaking abruptly.

In contrast, a different fracture pattern emerges when stretched in the *y* direction. Figure 7e–g show the lattice at 0%, 1%, and 9.0% strain, with no structural failure. Beyond the critical strain value (e.g., 9.8%), the lattice forms linear atomic chains (LACs) and undergoes some stages of plastic deformation. The stress accumulates primarily in the C–C bonds, forming 12-atom and 3-atom rings, leading to distinct fracture behavior.

Regarding the other extreme of the systems investigated here, NPG-9 is depicted in Figure 8, where the behavior observed in the case of *n* = 3 is evident with greater clarity. Panels 8a–d show NPG-9 at 0%, 1%, 11.8%, and 12.1% strain. When tension is in the *x* direction, the 3- and 12-membered rings contribute minimally to the system’s stress. In contrast, as shown in panels 8e–h, with strains of 0%, 1%, 7.5%, and 7.9%, there is stress primarily in these rings, but also in the hexagonal rings, confirming the more rigid response in the *y* direction and consequently making the system more susceptible to fracture (around 8%, compared to approximately 12% in the *x* direction).

It is essential to mention that the composition of the class of NPGs studied here is based on the pores generated by the insertion of linkers formed by pairs of trivalent sp^2^ carbon atoms between graphene nanoribbons. Thus, the increase in porosity is driven by two factors. First is the width of the nanoribbon: the smaller the width, the higher the pore density in the system. This factor was the focus of this study. Another possible way to increase porosity would be to increase the number of such pairs between the nanoribbons. In this second case, a behavior analogous to that previously investigated regarding the width of the nanoribbons is expected; however, with the presence of sp hybridization, it would differ from the situation studied here. Strain in the longitudinal direction of the nanoribbons primarily causes stress accumulation in the ribbons, while strain in the direction parallel to the arrangement of the carbon-bridge linkers mainly causes stress accumulation in these linkers since the nanoribbons have significantly fewer degrees of freedom due to the honeycomb arrangement of the atoms. Therefore, increasing the porosity by adding more linkers formed by pairs of trivalent sp^2^ carbon atoms would lead to the same behavior already reported for the NPGs of different widths investigated here.

All the NPGs investigated here were simulated with different initial velocity distributions, derived from a Gaussian distribution, to obtain statistical results and present the measurement errors, as shown in Table 1.

## Conclusions

The results of this study provide insights into the properties of a family of nanoporous graphene and its potential applications. Our investigation of this new 2D carbon allotrope, derived from AGNRs through lateral heterojunctions, highlighted its structural, stability, electronic, and mechanical characteristics. DFT and AIMD calculations confirm that NPG maintains high dynamical and thermal stability even at temperatures up to 1000 K, reassuring the audience about its potential in high-temperature environments. This stability indicates the material’s robustness under thermal stress, making it a promising candidate for applications where temperature fluctuations are a concern.

Structurally, incorporating 3-, 6-, and 12-membered carbon rings into the NPG lattice contributes to its electronic and mechanical properties. The material exhibits a formation energy that decreases with increasing AGNR width, suggesting enhanced stability for wider nanoribbons, and tends toward graphene-like cohesion energy. Thus, despite nanopores, NPG maintains a planar lattice without significant buckling, and the bond lengths are comparable to those found in graphene, except for the bonds involved in the triangular rings.

The dynamic stability of NPG, as verified by phonon dispersion curves, also demonstrates that the material is free from significant mechanical instabilities. These results are crucial for practical applications, indicating that NPG can withstand certain degrees of deformation without structural failure.

Regarding electronic structure, NPG exhibits metallic behavior, which excites the audience about its potential for efficient charge transport. This allows for efficient charge transport. Anisotropic conductance, with differing electronic properties along various crystallographic directions, offers opportunities for directional conductivity control in nanoelectronic devices. This feature, combined with the dominance of p-orbitals in the electronic density of states, reinforces the similarity of NPG to graphene in terms of its electronic behavior.

Finally, NPG exhibits a mechanical response with varying characteristics under deformation in the *x* and *y* directions. The material is less pliable under stress applied parallel to the length of the nanoribbon, showing increased susceptibility to tension in the *y* direction due to the rigidity present in the triangular ring. Under stress perpendicular to the length of the nanoribbon, fractures are more common in the heterojunction regions. The observed fracture patterns and stress–strain behavior highlight the anisotropic mechanical response of NPG, which varies with nanoribbon width and the direction of applied strain. Despite these variations, NPG’s Young’s modulus values, ranging from 394 to 690 GPa, indicate substantial stiffness, approximately 70% of the value presented for graphene.

## Supporting Information Available

The Supporting Information includes a discussion of the mechanical properties of the systems investigated here when deposited on a substrate (Section S1) and their response when the temperature is continuously changed (Section S2) (PDF)
