# Supplementary material for: Computational Design of 2D Nanoporous Graphene via Carbon-Bridged Lateral Heterojunctions in Armchair Graphene Nanoribbons
Source: ACS Omega. 2025 Apr 23;10(17):17159–69. doi: 10.1021/acsomega.4c07524 (PMC12059925; doi:10.1021/acsomega.4c07524)
Supplement: Supplementary file 1 — ao4c07524_si_001.pdf [file ao4c07524_si_001.pdf]

Supplementary Information for Computational Design of 2D Nanoporous Graphene via Carbon-Bridged Lateral Heterojunctions in Armchair Graphene Nanoribbons

Rodrigo A. F. Alves,[†,‡] Kleuton A. L. Lima,[†,‡] Daniel A. da Silva,[¶] Fábio L. L. Mendonça,[§,¶] Luiz A. Ribeiro Junior,[†,‡] and Marcelo L. Pereira Junior[*,§,‖]

†*University of Brasília, Institute of Physics, 70910900 Brasília, Federal District, Brazil.*

‡*Computational Materials Laboratory, LCCMat, Institute of Physics, University of Brasília, 70910900 Brasília, Federal District, Brazil.*

¶*Professional Postgraduate Program in Electrical Engineering (PPEE), Department of Electrical Engineering, College of Technology, University of Brasília, 70910900 Brasília, Federal District, Brazil.*

§*University of Brasília, College of Technology, Department of Electrical Engineering, 70910900 Brasília, Federal District, Brazil.*

‖*Rice University, Materials Science and NanoEngineering, 77005, Houston, Texas, United States.*

E-mail: marcelo.lopes@unb.br

S1 Mechanical Properties of NPGs Deposited on Substrate

To assess the influence of an additional external agent on the system (such as pressure and temperature), we employed calculations capable of describing the mechanical properties of the NPG systems studied here, deposited on a gold substrate instead of the gas phase used in the main text. For this purpose, the same parameters and systems initially used in the gas phase were repositioned at a distance of 5 Å from the edge of the box, which, besides being modified to a non-periodic configuration, now behaves as a potential surface modeled by the 12-6 Lennard-Jones (LJ) style,[1] given by the following expression:

$$E_{\text{substrate}} = 4\varepsilon_{\text{substrate}} \left[\left(\frac{\sigma_{\text{substrate}}}{r} \right)^{12} - \left(\frac{\sigma_{\text{substrate}}}{r} \right)^{6} \right], \quad (1)$$

where r is the distance between each carbon atom and the substrate, $\varepsilon_{\text{substrate}}$ represents the potential well depth, and $\sigma_{\text{substrate}}$ is the equilibrium distance between the atoms and the substrate. We also defined a cutoff radius. If $r > r_C$, then $E_{\text{substrate}} = 0$. To investigate the mechanical properties of NPG deposited on a gold substrate, we used $\varepsilon_{\text{substrate}} = 31.81$ meV and $\sigma_{\text{substrate}} = 2.99$ Å.[2]

Figure S1 presents the stress-strain curves for the NPG systems studied here. The elastic constants, such as Young's modulus (Y_M), critical stress (σ_C), and critical strain (ε_C), for both the gas phase system and the system deposited on a substrate, are provided in Table S1. The behavior of the stress-strain curves and the values obtained from these graphs have been discussed in detail in the main text. The main point to emphasize regarding considering systems deposited on a substrate is that this increases the stability and, mainly, the planarity of the deposited systems. Since all NPGs are completely planar, the elastic properties of the investigated systems were not altered, as can be observed primarily in Table S1.

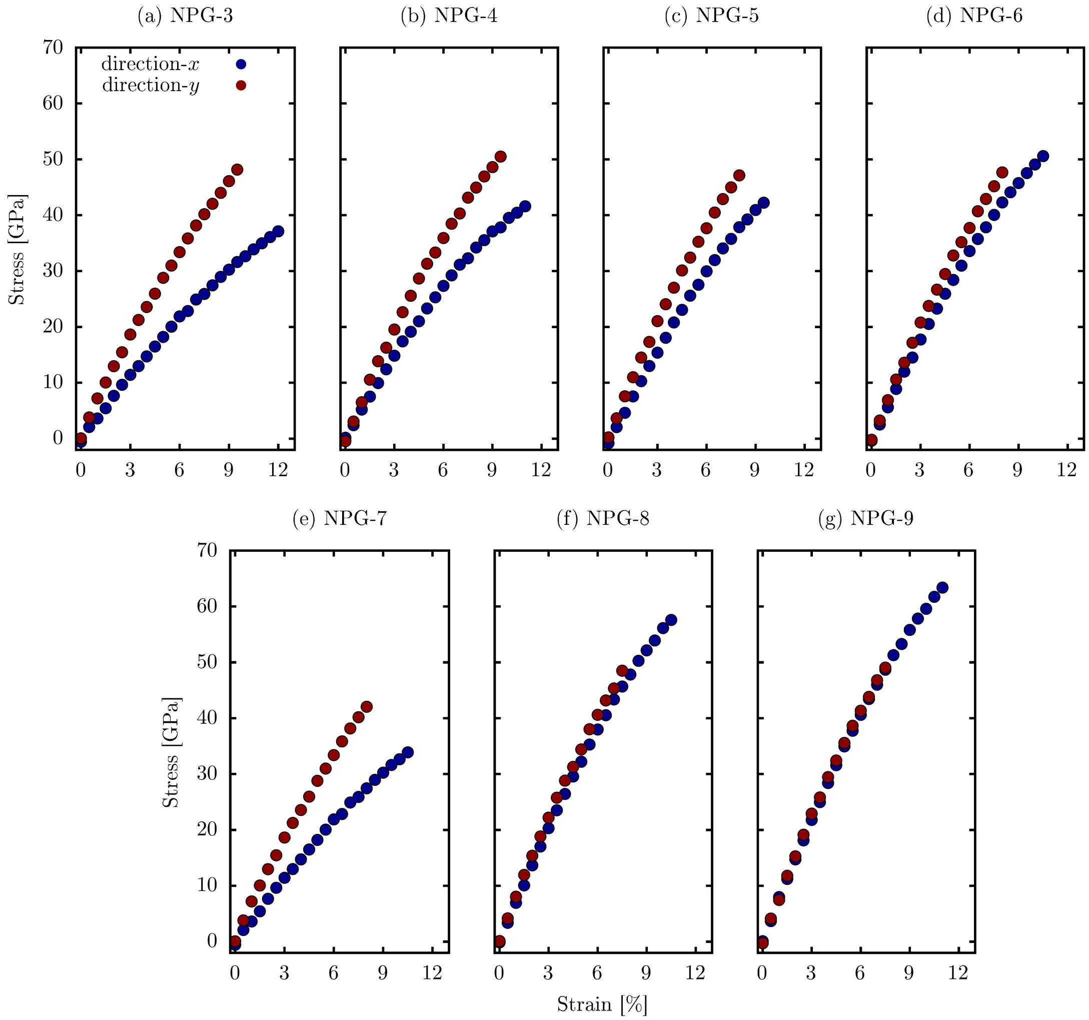

Figure S1: When deposited on a gold substrate, stress-strain curves for the NPG systems under uniaxial deformation in the x direction (blue) and the y direction (red).

Table S1: Elastic constants of NPG with and without Au substrate.

n	Without Substrate						With Substrate					
	Y_M (GPa)		σ_C (GPa)		ε_C (%)		Y_M (GPa)		σ_C (GPa)		ε_C (%)	
	x-dir	y-dir	x-dir	y-dir	x-dir	y-dir	x-dir	y-dir	x-dir	y-dir	x-dir	y-dir
3	394.4	619.3	37.7	48.4	12.3	9.8	395.0	631.7	37.9	49.3	12.4	9.8
4	498.7	693.0	42.1	49.1	11.1	9.0	499.4	702.7	50.4	42.9	9.5	9.7
5	557.6	718.2	42.2	49.7	9.3	8.7	563.4	719.8	42.9	47.2	9.7	8.1
6	595.1	729.1	53.9	49.5	11.6	8.4	620.4	726.2	53.6	48.7	11.5	8.3
7	650.9	756.7	56.6	50.0	10.9	8.1	674.2	771.6	57.2	49.3	11.1	7.8
8	665.8	754.2	61.0	49.8	11.6	7.9	694.0	760.9	58.3	48.6	10.7	7.6
9	685.8	758.1	65.3	50.6	11.8	7.9	740.8	768.0	63.6	50.1	11.1	7.8

S2 NPG Stability as a temperature function

In evaluating the stability dependence of the NPGs, after equilibrating and thermalizing the systems at room temperature and zero pressure, we applied an increase in the thermal bath temperature for 500 ps at a rate of 15.4 K/ps, using a canonical ensemble (NVT), ranging from 300 K to 8000 K. According to the first law of thermodynamics, $\mathrm{d}U = \delta Q - p\mathrm{d}V$, the

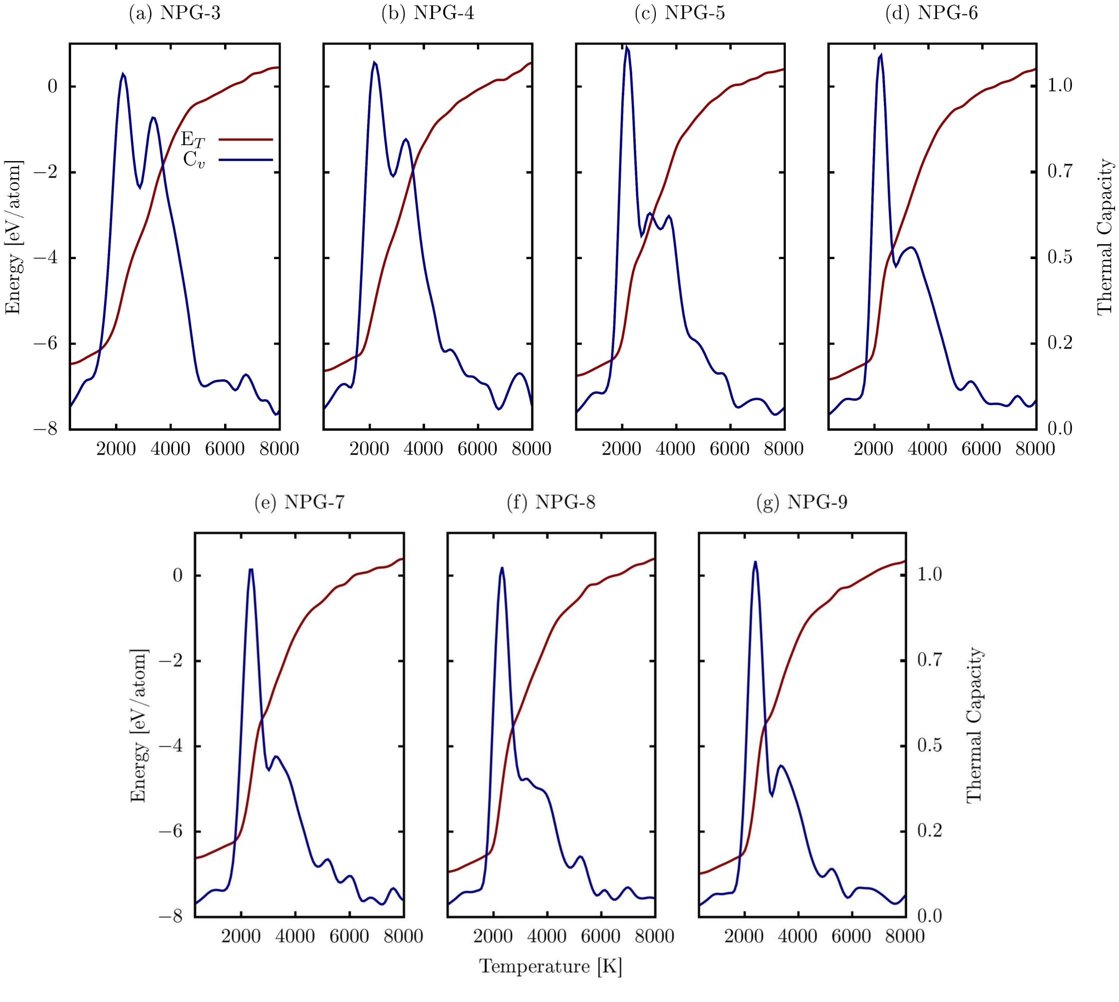

Figure S2: Total energy (red) and heat capacity (blue) curves as a function of temperature for NPGs obtained from 3- to 9-AGNR.

internal energy is expressed as a function of temperature and volume as follows:

$$\delta Q = \left(\frac{\partial U}{\partial T}\right)_V dT + \left(\frac{\partial U}{\partial V}\right)_T dV + pdV. \quad (2)$$

In the canonical ensemble, the volume is constant, so we have $C_V = \delta Q/dT = (\partial U/\partial T)_V$. Figure S2 illustrates the evolution of total energy as a function of temperature (in red), heat capacity (in blue), and temperature. Our results reveal that increasing the width of the nanoribbons used to fabricate the nanoporous systems does not influence their temperature-dependent stability. This outcome is attributed to the connection between the linkers formed by pairs of trivalent sp^2 carbon atoms and the nanoribbons being less stable than the linker structure and the nanoribbons. Therefore, the melting point at 2338 K is associated with the nanoribbon and remains constant across all NPGs investigated, independent of width.

It is important to note that this melting point was defined as the moment of the most significant energy change in the system. However, the melting process begins around 1900 K. It continues up to 6000 K, involving multiple bond breakages and forming linear atomic chains (LACs), as shown for other carbon allotropes before.[3]

References

(1) Yang, J.; Chen, Y.; Yang, Z.; Dai, L.; Choi, H.; Meng, Z. Unveiling the Nanoconfinement Effect on Crystallization of Semicrystalline Polymers Using Coarse-Grained Molecular Dynamics Simulations. *Polymers* **2024**, *16*, 1155.

(2) Brann, M. R.; Hansknecht, S. P.; Ma, X.; Sibener, S. Differential condensation of methane isotopologues leading to isotopic enrichment under non-equilibrium gas–surface collision conditions. *The Journal of Physical Chemistry A* **2021**, *125*, 9405–9413.

(3) Pereira, M.; Da Cunha, W.; De Sousa, R.; Nze, G. A.; Galvão, D.; Ribeiro, L. On the mechanical properties and fracture patterns of the nonbenzenoid carbon allotrope

(biphenylene network): a reactive molecular dynamics study. *Nanoscale* **2022**, *14*, 3200–3211.
